# Toward Male Individualization with Rapidly Mutating Y-Chromosomal Short Tandem Repeats

**DOI:** 10.1002/humu.22599

**Published:** 2014-06-10

**Authors:** Kaye N Ballantyne, Arwin Ralf, Rachid Aboukhalid, Niaz M Achakzai, Maria J Anjos, Qasim Ayub, Jože Balažic, Jack Ballantyne, David J Ballard, Burkhard Berger, Cecilia Bobillo, Mehdi Bouabdellah, Helen Burri, Tomas Capal, Stefano Caratti, Jorge Cárdenas, François Cartault, Elizeu F Carvalho, Monica Carvalho, Baowen Cheng, Michael D Coble, David Comas, Daniel Corach, Maria E D'Amato, Sean Davison, Peter de Knijff, Maria Corazon A De Ungria, Ronny Decorte, Tadeusz Dobosz, Berit M Dupuy, Samir Elmrghni, Mateusz Gliwiński, Sara C Gomes, Laurens Grol, Cordula Haas, Erin Hanson, Jürgen Henke, Lotte Henke, Fabiola Herrera-Rodríguez, Carolyn R Hill, Gunilla Holmlund, Katsuya Honda, Uta-Dorothee Immel, Shota Inokuchi, Mark A Jobling, Mahmoud Kaddura, Jong S Kim, Soon H Kim, Wook Kim, Turi E King, Eva Klausriegler, Daniel Kling, Lejla Kovačević, Leda Kovatsi, Paweł Krajewski, Sergey Kravchenko, Maarten H D Larmuseau, Eun Young Lee, Ruediger Lessig, Ludmila A Livshits, Damir Marjanović, Marek Minarik, Natsuko Mizuno, Helena Moreira, Niels Morling, Meeta Mukherjee, Patrick Munier, Javaregowda Nagaraju, Franz Neuhuber, Shengjie Nie, Premlaphat Nilasitsataporn, Takeki Nishi, Hye H Oh, Jill Olofsson, Valerio Onofri, Jukka U Palo, Horolma Pamjav, Walther Parson, Michal Petlach, Christopher Phillips, Rafal Ploski, Samayamantri P R Prasad, Dragan Primorac, Gludhug A Purnomo, Josephine Purps, Hector Rangel-Villalobos, Krzysztof Rębała, Budsaba Rerkamnuaychoke, Danel Rey Gonzalez, Carlo Robino, Lutz Roewer, Alexandra Rosa, Antti Sajantila, Andrea Sala, Jazelyn M Salvador, Paula Sanz, Cornelia Schmitt, Anil K Sharma, Dayse A Silva, Kyoung-Jin Shin, Titia Sijen, Miriam Sirker, Daniela Siváková, Vedrana Škaro, Carlos Solano-Matamoros, Luis Souto, Vlastimil Stenzl, Herawati Sudoyo, Denise Syndercombe-Court, Adriano Tagliabracci, Duncan Taylor, Andreas Tillmar, Iosif S Tsybovsky, Chris Tyler-Smith, Kristiaan J van der Gaag, Daniel Vanek, Antónia Völgyi, Denise Ward, Patricia Willemse, Eric PH Yap, Rita YY Yong, Irena Zupanič Pajnič, Manfred Kayser

**Affiliations:** 1 Department of Forensic Molecular Biology, Erasmus MC University Medical Centre RotterdamRotterdam, The Netherlands; 2 Office of the Chief Forensic Scientist, Victoria Police Forensic Services DepartmentMacleod, Victoria, Australia; 3 Forensic Genetic Unit, Immunology and Biochemistry Laboratory, Faculty of Sciences, Mohammed V Agdal UniversityRabat, Morocco; 4 DNA Typing Laboratory, Centre of Excellence in Molecular Biology, CEMB, University of the PunjabLahore, Pakistan; 5 Forensics Genetic Service, Central Delegation, National Institute of Legal Medicine and Forensic Sciences, I.PCoimbra, Portugal; 6 The Wellcome Trust Sanger Institute, Wellcome Trust Genome CampusHinxton, South Cambridgeshire, UK; 7 Institute of Forensic Medicine, Faculty of Medicine, University of LjubljanaLjubljana, Slovenia; 8 Department of Chemistry, University of Central FloridaOrlando, Florida; 9 National Center for Forensic Science, University of Central FloridaOrlando, Florida; 10 King's College London, Department of Forensic and Analytical ScienceLondon, UK; 11 Institute of Legal Medicine, Innsbruck Medical UniversityInnsbruck, Austria; 12 Servicio de Huellas Digitales Genéticas, Facultad de Farmacia y Bioquímica, Universidad de Buenos Aires and CONICETBuenos Aires, Argentina; 13 Institute of Legal Medicine, University of ZurichZurich, Switzerland; 14 Department of Forensic Genetics, Institute of Criminalistics PraguePrague, Czech Republic; 15 Department of Public Health Sciences and Pediatrics, University of TurinTurin, Italy; 16 Forensic Genetics Unit, Institute of Legal Medicine, University of Santiago de CompostelaSantiago de Compostela, Spain; 17 Service de Genetique, Site Centre Hospitalier Felix GuyonSaint-Denis, Reunion; 18 DNA Laboratory, Department of Ecology, Biology Institute, State University of Rio de JaneiroRio de Janeiro, Brazil; 19 Institute of Forensic Science, Yunnan Provincial Department of Public SecurityKunming, China; 20 National Institute of Standards and Technology, Applied Genetics GroupGaithersburg, Maryland; 21 Institut de Biologia Evolutiva (CSIC-UPF), Departament de Ciències Experimentals i de la Salut, Universitat Pompeu FabraBarcelona, Spain; 22 Forensic DNA Lab, Department of Biotechnology, University of the Western CapeCape Town, South Africa; 23 Department of Human Genetics, Leiden University Medical CenterLeiden, The Netherlands; 24 DNA Analysis Laboratory, Natural Sciences Research Institute, University of the PhilippinesDiliman, Quezon City, Philippines; 25 Laboratory of Forensic Genetics and Molecular Archaeology, Department of Imaging and PathologyKU Leuven, Leuven, Belgium; 26 Department of Forensic Medicine, Wrocław Medical UniversityWrocław, Poland; 27 Department of Family Genetics, The Norwegian Institute of Public HealthOslo, Norway; 28 Department of Forensic Medicine and Toxicology, Faculty of Medicine, University of BenghaziBenghazi, Libya; 29 Department of Forensic Medicine, Medical University of GdanskGdansk, Poland; 30 Human Genetics Laboratory, University of MadeiraFunchal, Portugal; 31 Department of Human Biological Traces (R&D), Netherlands Forensic InstituteThe Hague, The Netherlands; 32 Institut für Blutgruppenforschung LGC GmbHCologne, Germany; 33 Unidad de Genética Forense, Sección de Bioquímica del Departamento de Ciencias Forenses, Poder Judicial, San Joaquín de FloresHeredia, Costa Rica; 34 Department of Clinical and Experimental Medicine, Faculty of Health Sciences, Linköping UniversityLinköping, Sweden; 35 Department of Forensic Genetics and Forensic Toxicology, National Board of Forensic MedicineLinköping, Sweden; 36 Department of Legal Medicine, Faculty of Medicine, University of TsukubaTsukuba City, Japan; 37 Institute of Legal Medicine, Martin-Luther University HalleHalle, Germany; 38 National Research Institute of Police ScienceKashiwa, Japan; 39 Department of Genetics, University of LeicesterLeicester, UK; 40 DNA Forensic Division, Supreme Prosecutors’ OfficeSeoul, South Korea; 41 DNA Analysis Laboratory, Eastern District Office, National Forensic ServiceGangwon-do, South Korea; 42 Department of Biological Sciences, Dankook UniversityCheonan, South Korea; 43 Institute of Legal Medicine, University of SalzburgSalzburg, Austria; 44 Institute for Genetic Engineering and BiotechnologySarajevo, Bosnia and Herzegovina; 45 Laboratory of Forensic Medicine and Toxicology, School of Medicine, Aristotle University of ThessalonikiThessaloniki, Greece; 46 Department of Forensic Medicine, Centre for Biostructure, Medical University of WarsawWarsaw, Poland; 47 Department of Human Genomics, Institute of Molecular Biology and Genetics NASUKiev, Ukraine; 48 Department of Forensic Medicine, Yonsei University College of MedicineSeoul, South Korea; 49 Genomac Forensic InstitutePrague, Czech Republic; 50 Laboratório de Genética Aplicada, Departamento de Biologia, Universidade de AveiroAveiro, Portugal; 51 Section of Forensic Genetics, Department of Forensic Medicine, Faculty of Health and Medical Sciences, University of CopenhagenCopenhagen, Denmark; 52 Central Forensic Science Laboratory, Directorate of Forensic Science Services, Ministry of Home Affairs, Government of IndiaKolkata, India; 53 Laboratory of DNA Fingerprinting Services, Centre for DNA Fingerprinting and DiagnosticsHyderabad, India; 54 School of Forensic Medicine, Kunming Medical UniversityKunming, China; 55 DNA and Biology Unit, Central Scientific Crime Detection Division, Office of Forensic Science Police, Royal Thai PoliceBangkok, Thailand; 56 Section of Legal Medicine, Department of Biomedical Science and Public Health, Università Politecnica delle MarcheAncona, Italy; 57 Department of Forensic Medicine, Hjelt Institute, University of HelsinkiHelsinki, Finland; 58 DNA Laboratory, Institute of Forensic Medicine, Network of Forensic Science Institutes, Budapest, Ministry of Public Administration and JusticeBudapest, Hungary; 59 Eberly College of Science, The Pennsylvania State UniversityUniversity Park, Pennsylvania; 60 Department of Medical Genetics, Centre for Biostructure, Medical University of WarsawWarsaw, Poland; 61 The Henry C. Lee College of Criminal Justice and Forensic Sciences, University of New HavenWest Haven, Connecticut; 62 University of Split, Medical School, Split and University of Osijek, Medical SchoolOsijek, Croatia; 63 Eijkman Institute for Molecular BiologyJakarta, Indonesia; 64 Institute of Legal Medicine and Forensic Sciences, Charité – Universitätsmedizin BerlinBerlin, Germany; 65 Instituto de Investigación en Genética Molecular, Universidad de Guadalajara (CUCienega-UdeG)Jalisco, México; 66 Department of Pathology, Faculty of Medicine Ramathibodi Hospital, Mahidol UniversityBangkok, Thailand; 67 Medical Sciences Unit, Center of Life Sciences, University of MadeiraFunchal, Portugal; 68 Institute of Applied Genetics, Department of Molecular and Medical Genetics, University of North Texas Health Science CenterFort Worth, Texas; 69 Institute of Legal Medicine, Faculty of Medicine, University of CologneCologne, Germany; 70 Department of Anthropology, Comenius University in BratislavaBratislava, Slovakia; 71 DNA Laboratory, Genos LtdZagreb, Croatia; 72 Facultad de Microbiología, Universidad de Costa Rica, San Pedro de Montes de OcaSan José, Costa Rica; 73 Forensic Science South Australia, Adelaide, Australia and School of Biological Sciences, Flinders UniversityAdelaide, Australia; 74 Scientific and Practical Centre of the State Committee of Forensic ExpertisesMinsk, Belarus; 75 Forensic DNA ServicePrague, Czech Republic; 76 2nd Medical Faculty, Institute of the Legal Medicine, Charles UniversityPrague, Czech Republic; 77 Forensic Science South AustraliaAdelaide, Australia; 78 Defence Medical and Environmental Research Institute, DSO National LaboratoriesSingapore

**Keywords:** Y-chromosome, Y-STRs, haplotypes, RM Y-STRs, paternal lineage, forensic

## Abstract

Relevant for various areas of human genetics, Y-chromosomal short tandem repeats (Y-STRs) are commonly used for testing close paternal relationships among individuals and populations, and for male lineage identification. However, even the widely used 17-loci Yfiler set cannot resolve individuals and populations completely. Here, 52 centers generated quality-controlled data of 13 rapidly mutating (RM) Y-STRs in 14,644 related and unrelated males from 111 worldwide populations. Strikingly, >99% of the 12,272 unrelated males were completely individualized. Haplotype diversity was extremely high (global: 0.9999985, regional: 0.99836–0.9999988). Haplotype sharing between populations was almost absent except for six (0.05%) of the 12,156 haplotypes. Haplotype sharing within populations was generally rare (0.8% nonunique haplotypes), significantly lower in urban (0.9%) than rural (2.1%) and highest in endogamous groups (14.3%). Analysis of molecular variance revealed 99.98% of variation within populations, 0.018% among populations within groups, and 0.002% among groups. Of the 2,372 newly and 156 previously typed male relative pairs, 29% were differentiated including 27% of the 2,378 father–son pairs. Relative to Yfiler, haplotype diversity was increased in 86% of the populations tested and overall male relative differentiation was raised by 23.5%. Our study demonstrates the value of RM Y-STRs in identifying and separating unrelated and related males and provides a reference database.

## Introduction

Genetic characterization of male individuals and populations by means of Y-chromosome DNA polymorphisms is relevant in various fundamental and applied areas of human genetics such as in evolutionary genetics and population history, for example, for modeling global and regional human evolution, mapping migration patterns across the globe, and tracking cultural and demographic factors such as patrilocality, extrapair paternity, endogamy, and polygyny. Further, Y-chromosome DNA analysis is important in genetic genealogy and for community genetic purposes such as personal ancestry identification, as well as for the identification of male lineages and inferring paternal genetic ancestry for judicial and investigative purposes [Kayser et al., 1997; Underhill et al., 2000; Hammer et al., 2001; Oota et al., 2001; Jobling and Tyler-Smith, 2003; Roewer et al., 2005; Shi et al., 2010]. Similarities at Y-chromosome DNA markers are usually interpreted as indicating shared paternal ancestry of individuals and populations, whereas differences are used to conclude the absence of close paternal relationships. Such interpretations, however, depend in part on the underlying mutation rates of the Y-DNA markers used. Slowly evolving Y-chromosomal single-nucleotide polymorphisms (Y-SNPs), with an average mutation rate of about 3 × 10^−8^ per nucleotide per generation [Xue et al., 2009; Poznik et al., 2013], are especially suitable for studying distant relationships between male individuals and populations [Underhill et al., 2000; van Oven et al., 2014]. More quickly evolving Y-chromosomal short tandem repeat (Y-STRs) polymorphisms, also referred to as Y-microsatellites, with an average mutation rate of about 10^−3^ per locus per generation [Goedbloed et al., 2009; Ballantyne et al., 2010], have proven useful for testing short to medium timescale paternal relationships [Kayser et al., 2003; Kayser et al., 2005; Roewer et al., 2005; Coble et al., 2009; van Oven et al., 2011] such as enabling male lineages to be connected through common paternal ancestry [Coble et al., 2009], for the same lineages be separated and individualized within that shared ancestral lineage [King and Jobling, 2009], and for the origins of entire population groups be elucidated [Parkin et al., 2007; Rębała et al., 2007]. Forensic usage of Y-STRs has largely focused on identifying paternal lineages using a core set of markers, linking suspects, and crime samples for investigation purposes [Kayser et al., 1997; Roewer, 2009].

Haplotypes generated from conventional Y-STRs, such as the widely used 17 markers included in the commercially available AmpFlSTR® Yfiler® PCR Amplification Kit (Life Technologies, San Francisco, CA) (subsequently referred to as Yfiler), suffer from two main limitations: (1) their inability to conclusively resolve some male lineages due to identical haplotypes arising in individuals that are not of common descent because of recurrent mutation, and (2) their inability to differentiate between paternally related males due to the moderately low mutation rate of the loci tested. In general, Y-STRs with much higher mutation rates than those of the conventionally used loci are expected to overcome or at least reduce both limitations. In a previous comprehensive Y-STR mutation rate study where nearly 200 Y-STRs were investigated in almost 2,000 father–son pairs confirmed by autosomal DNA analysis [Ballantyne et al., 2010], 13 Y-STRs with exceptionally high (>10^−2^ per locus per generation) mutation rates were identified and termed rapidly mutating (RM) Y-STRs. Furthermore, in this previous study and a subsequent study [Ballantyne et al., 2012], theoretical and the first empirical evidence were provided to show that this set of 13 RM Y-STRs is able to achieve an order of magnitude higher male relative differentiation than is available with the commonly used Yfiler set, as well as to drastically improve male lineage differentiation over Yfiler.

However, to fully explore the potential for the RM Y-STR set in differentiating unrelated as well as related males for various purposes, much more data are needed. Therefore, the International RM Y-STR Study Group, a worldwide collaboration between 52 laboratories, was formed. Group members, chosen based on pre-existing practical experience in Y-STR analysis, genotyped under quality-controlled conditions the 13 RM Y-STRs in 14,644 males including 12,272 unrelated males from 111 worldwide populations and 4,744 closely related males. To compare the RM Y-STR set with conventional Y-STRs, Yfiler data were gathered in a subset of 7,784 unrelated men from 65 worldwide populations as well as in most of the male relatives analyzed for RM Y-STRs. The large collection of RM Y-STR haplotypes presented and explored in the present study enables a better understanding of their value to differentiate related and unrelated male individuals and populations, and additionally provides a suitable reference database for future use of RM Y-STRs in forensic, genealogical, anthropological, and population genetic studies.

## Materials and Methods

### DNA Samples

With a worldwide coverage in mind, contributing laboratories were identified and recruited based on their previous submission of Y-STR population data to the Y-chromosome Haplotype Reference Database (YHRD; http://www.yhrd.org), hence having proven experience in Y-STR analysis. Each of the 52 contributing laboratories within the International RM Y-STR Study Group genotyped a selection of their own in-house population sample sets, each consisting of seven to 634 individuals (median 100 individuals per population sample) across 111 defined population samples per laboratory. Population sets were to include only unrelated males. Three population samples were notable exceptions—the Biaka Pygmy group and the Bhutanese Lhokpu and Mönpa language groups. These populations, and particular sample sets used here, are known to contain high numbers of male relatives (Biaka), or were specifically selected as having extremely low resolution with Yfiler (Bhutan). Because these samples were ascertained differently to the other 108 populations within the study, they were excluded from the continental groups during analysis to avoid bias. The Aboriginal Australian samples used here were also not ascertained completely randomly, as they were selected based on Y-SNP haplogroups known to be authentic for Aboriginal Australians to avoid admixture effects as described elsewhere [Taylor et al. 2012].

A subset of 7,784 individuals from 65 populations were additionally genotyped with the AmpF*l*STR Yfiler PCR Amplification Kit (Life Technologies) for the most commonly used panel of 17 Y-STRs. Relative pairs were also analyzed, with 2,339 newly genotyped father–son pairs, 30 brother pairs, and three uncle–nephew pairs, for which relationship had previously been confirmed by autosomal DNA analysis; this dataset of male relatives was supplemented by 156 previously published relative pairs [Ballantyne et al., 2012].

### Y-STR genotyping

The organizing laboratory (Department of Forensic Molecular Biology, Erasmus MC University Medical Centre Rotterdam) provided genotyping protocols, allelic ladders, and tools for allele calling to all participating laboratories, and organized a quality control exercise prior to population data generation (for details see below). The 13 single- or multicopy RM Y-STR markers (DYF387S1, DYF399S1, DYF403S1, DYF404S1, DYS449, DYS518, DYS526, DYS547, DYS570, DYS576, DYS612, DYS626, and DYS627) were amplified in three multiplex PCRs, as described in Supp. Tables S1 and S2. PCR-amplified products were separated and detected using participating laboratories’ standard protocols for analyzing STRs—in either ABI310, ABI3100, ABI3130, ABI3500, or ABI3730 Genetic Analyzers with POP-4, POP-6, or POP-7, and size standards ILS-600, ILS-500, or Genescan 500-LIZ. Allele calling was performed with GeneMapper (Life Technologies) or GeneMarker (SoftGenetics, State College, PA) utilizing custom panel and bin sets. The participating laboratories used their own primers and other consumables (except allelic ladders as provided by the host).

The commercially available AmpFlSTR® Yfiler® PCR Amplification Kit (Life Technologies) targeting 17 conventional Y-STRs was used and allele calling was performed according to the manufacturer's instructions.

### Genotyping Quality Control

To ensure genotyping consistency between the laboratories, all participants received allelic ladders prepared by the organizing laboratory, and six blind control DNA samples previously genotyped by the organizing laboratory. Genotyping of population samples was only allowed after a participating laboratory demonstrated the correct genotyping of these six blind control DNA samples at all 13 RM Y-STRs. If a participant reported erroneous genotypes, screenshots were requested and submitted to the organizing laboratory for an evaluation of the possible cause of error(s). Once the cause of error was identified, three additional blind control DNA samples were provided and genotyped by the participant. If these samples were typed correctly, the laboratory was allowed to type their population samples. In cases of unexpected results such as previously unknown alleles or microvariants, screenshots were requested and submitted to the organizing laboratory for inspection. Independent genotyping, and in some case DNA sequencing, was performed to resolve differences in genotyping of certain samples. Samples with missing data from more than one marker were excluded from data analysis to prevent low quality samples affecting genotype and haplotype distributions. Further, any differences observed between relative pairs were confirmed through duplicate, independent PCR amplifications, and genotyping, and in some cases, DNA sequencing.

Additional quality control was performed for the multicopy markers DYF403S1a+b and DYF399S1, whereby a subset of population sample electropherograms for these markers were sent to the organizing laboratory by all participants for blind confirmatory genotype scoring.

### RM Y-STR Nomenclature

The nomenclature of the RM Y-STRs was updated to comply with the guidelines of the International Society of Forensic Genetics—ISFG [Gusmão et al., 2006], and to incorporate new variation observed in repeat structures. As such, all data collated were translated to comply with the updated nomenclature. Supp. Table S3 shows the repeat structure and allele designations used during the project, determined in collaboration with Life Technologies and L. Gusmao (IPATIMUP, Porto, Portugal). The RM Y-STR nomenclature used in the present study is in agreement with that used in the Powerplex® Y23 kit (Promega, Madison, WI) and in the Yfiler Plus Y-STR kit (Life Technologies) for the RM Y-STR markers included, respectively.

### Data Analysis

Haplotype diversities, average number of differing loci between pairs of individuals, pairwise *F*_ST_ genetic distances, and analysis of molecular variance (AMOVA) were calculated with Arlecore v3.5.1.3 [Excoffier and Lischer, 2010]. Theta (θ) values were calculated as per the reference Weir and Hill (2002). Molecular relationships between samples were analyzed using Network v4.6.1.1., applying the median-joining method. Weighting was applied as described elsewhere [Qamar et al., 2002], although based on updated mutation rates from Ballantyne et al. (2010) and http://www.yhrd.org. Only single-copy markers were utilized in network construction, due to the inability to assign multicopy genotypes to individual loci. Testing of statistical significance (*t*-tests) was performed in SPSS v17.0. Theoretical estimates for the rates of relative differentiation for each Y-STR panel were calculated using the formula *P*(*k* < 0) = 1 − *P*(*k* = 0) = 1 − *e*^−*Nm*^ [Ballantyne et al., 2010], where *N* represents the number of markers and *m* represents the average mutation rate of the set of markers obtained from the sampling from the posterior distribution.

## Results and Discussion

### Global Diversity of RM Y-STR Haplotypes and Male Lineage Differentiation

The RM Y-STR set applied here generated exceptionally high haplotype diversity in the worldwide sample set analyzed with 12,156 unique (i.e., not matching any other individual in this dataset) haplotypes observed in 12,272 unrelated samples from 111 worldwide populations (Table 1). The global haplotype diversity estimate was 0.9999985 (sampling variance = 2.85 × 10^−8^). Of the 102 haplotypes that were nonunique within this dataset (i.e., matched other individuals in this dataset), 90 were shared between two males, 10 between three males, and two between four males. All nonunique haplotypes were shared between males of the same geographic region, and all but six were from the same sampling population. The six nonunique haplotypes shared between different populations involved males from Argentina (Chubut and Rio Negro regions), Greenland (from two separate samples of Inuit), Czech Republic (from two sample sets), Cologne and Warsaw, and Bhutan (the Lhokpu and Mönpa tribes), respectively. As a result, the proportion of haplotypes shared between populations was exceptionally low at 0.05%, and the proportion of haplotypes shared within populations was slightly higher at 0. 8%.

**Table 1:** RM Y-STR Haplotype Characteristics in a Global Set of 12,272 Individuals from 111 Populations Summarized for Regional Groups

Group	Individuals	Populations	Haplotypes	Haplotype diversity	θ	Average number of differing loci
Sub-Saharan Africa	303	5	300	0.99993	0.00006	16.5
North Africa	452	4	445	0.99992	0.00010	16.2
Middle East	100	1	100	1.0	0	15.26
Central Asia	86	1	80	0.99836	0.0012	16.18
South Asia	661	8	644	0.99992	0.00009	17.5
East Asia	967	7	964	0.999994	0.000006	17.3
South East Asia	634	6	630	0.99998	0.00002	16.6
Aboriginal Australian	100	1	96	0.99919	0.00069	16.8
Native American	365	10	357	0.99986	0.00015	17.4
Admixed Native American	764	12	758	0.99998	0.00002	17.5
European	5,618	38	5599	0.9999988	0.000001	17.4
Migrant Sub-Saharan Africa	663	5	659	0.99998	0.000004	16.3
Migrant European	731	3	730	0.999996	0.000004	17.0
Migrant Asian	649	7	645	0.99998	0.00002	16.7
Bhutan^a^	78	2	56	0.99434	0.00794	13.9
Biaka Pygmy^a^	101	1	94	0.99822	0.00226	17.5
Global	12,272	111	12,156	0.9999985	0.00000238	16.5

a Bhutan and Biaka Pygmies were not assigned to a larger geographic group as the individuals sampled were ascertained differently (Bhutan), or are known to have different population characteristics to all other study populations (Biaka, see *Materials and Methods* and *DNA Samples*).

Within each regional geographic group (see Table 1), similar high levels of haplotype diversity were obtained ranging from 0.9999988 and 0.999996 in Europeans and Migrant Europeans (i.e., European populations sampled in North America and Australia), respectively, down to 0.99836 in Central Asians (although the sample size of the latter group was much lower than that of all other regional groups studied) (Table 1). The average number of differing loci was highest with 17.5 loci in Admixed Native Americans and South Asians, respectively, as well as with 17.4 loci in Native Americans and Europeans, respectively, and was lowest with 15.26 loci in Middle Easterners (Table 1). As the maximal possible number is 21, these results illustrate how strikingly different the haplotypes were within each of the regional groups.

At the population level (Supp. Table S4), similar high levels of haplotype diversity were observed across all populations tested. Of the 111 populations, 67 (60%) displayed haplotype diversities of 1.0, meaning that every individual tested per population (sample size ranging from seven to 467 in the various populations) had a different haplotype. The remaining populations had haplotype diversities ranging from 0.972 (Wichi, Salta Province, Argentina) to 0.999994 (Cologne, population 2).

Due to its highly multicopy nature, DYF403S1a+b caused concerns with some study group members regarding genotyping accuracy, so that additional quality control was performed for this marker (as well as for DYF399S1), as described in *Materials and Methods* section. There are four separate loci at the tetranucleotide DYF403S1a+b, three of which overlap in size. Differences in repeat sequences between the copies lead to the presence of partial (0.1, 0.2, and 0.3) alleles, which can challenge interpretation even in single source samples due to a lack of single base resolution with POP-4 during capillary electrophoresis. To determine the effect of removing this potentially problematic marker from the RM Y-STR panel, the entire dataset was additionally analyzed without DYF403S1a+b. The global haplotype diversity slightly decreased from 0.9999985 with the full 13-loci RM Y-STR set to 0.9999981 with the 12-loci set. This translated to an increase in the number of nonunique haplotypes from 116 to 152 in the 12,272 samples—a decrease of zero to five haplotypes per regional population, with an average of 0.32 fewer haplotypes within each of the 111 populations. Populations most affected by the omission of DYF403S1a+b were Aboriginal Australians (from 96 to 92 haplotypes among the 100 men tested), and Bhutan (from 56 to 51 haplotypes among the 78 men tested). However, the vast majority of the populations tested (90 of 111, 81.1%) were not affected by the removal of DYF403S1a+b, with the haplotype diversities remaining the same. Therefore, and because of the extra care in quality control employed for this marker, all following data analyses were performed based on the complete 13-loci RM Y-STR set.

### Genetic–Geographic Population Substructure with RM Y-STRs

The θ values for all regional groups and for almost all populations were exceptionally low (Table 1; Supp. Table S4), indicating relatively little population substructure detected with the RM Y-STR set. Indeed, AMOVA based on *F*_ST_ values (the inability to accurately assign the alleles of the multicopy markers to specific loci prevented *R*_ST_ values being calculated) demonstrated that 99.98% of haplotype variation was within populations, extremely high for Y-chromosome markers [Willuweit and Roewer, 2007], whereas 0.02% was among populations within the same regional group, and only 0.002% among regional groups. The global *F*_ST_ was as low at 0.00017. Between the regional groups, the average pairwise *F*_ST_ value was only 0.000127, with a maximum value of 0.00058 observed between Aboriginal Australians and Middle Eastern populations. Even when considering individual populations, the highest pairwise *F*_ST_ value observed was only 0.02815 between the Bhutan Lhokpu and Argentinian Wichi from Salta Province (data not shown). Across all population comparisons, an average pairwise *F*_ST_ value of 0.000826 was obtained.

**Figure 1:**
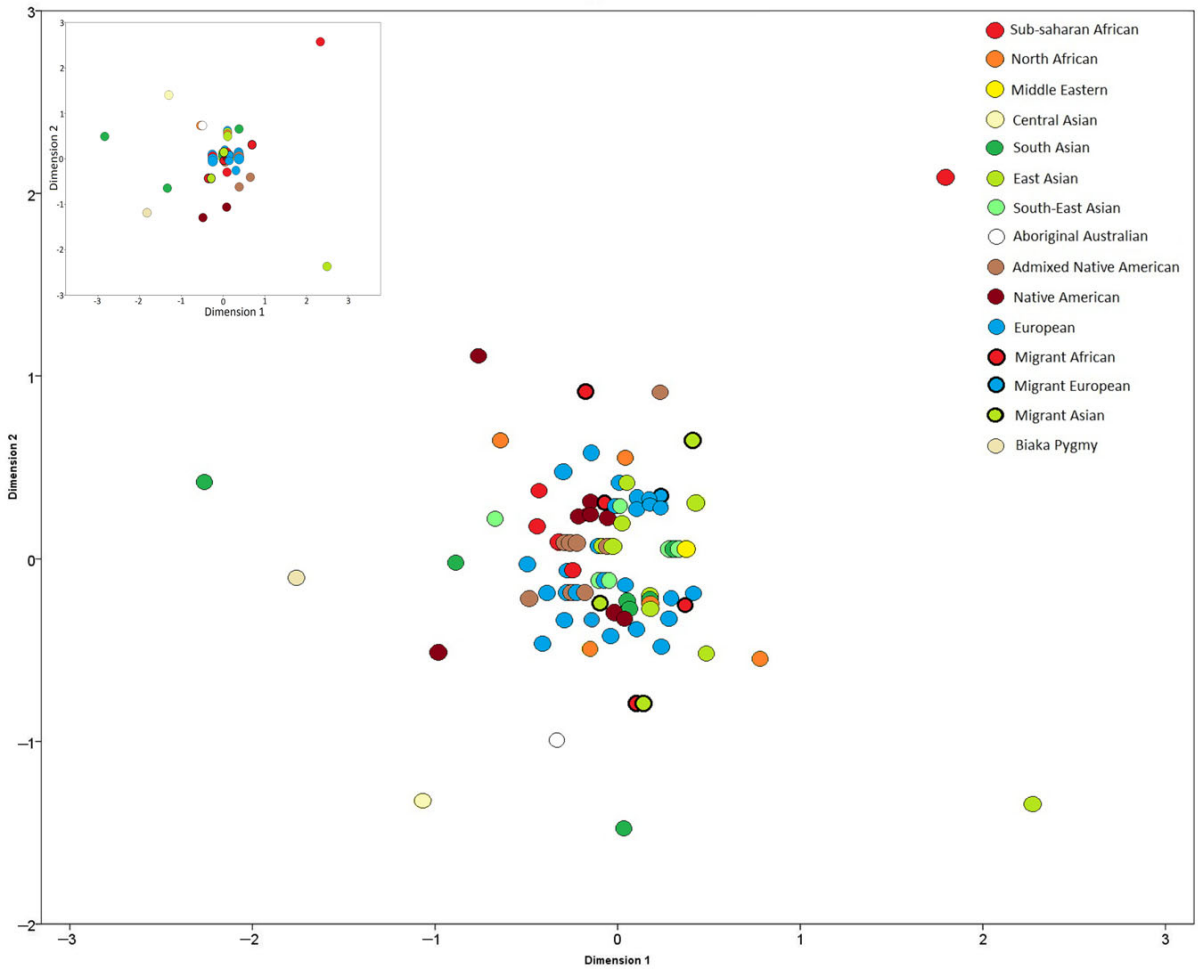
Two-dimensional plot of MDS analysis of Slatkin's linearized *F_ST_* values for RM Y-STR haplotypes in a global sample of 12,272 individuals from 111 populations (stress 0.07462). Smaller inset MDS shows the effect of equalized sample size (*N* = 100 individuals or fewer per population, stress = 0.02416).

To illustrate the magnitude of haplotypic differences between populations and geographic areas, multidimensional scaling (MDS) analysis was performed on Slatkin's linearized *F*_ST_ values (Fig. 1). The majority of the populations formed a loose cluster, with moderate dispersion across both dimensions. While there were several outlier populations (for example Pakistani Punjabis, Japanese Gunma, and Angola Kimbundos), overall there was no geographic pattern emerging, in line with the AMOVA results. It is notable that populations from the same geographic group did not necessarily cluster together (Fig. 1). The differences in population sample size (Supp. Table S4) did not greatly affect the dispersion of populations—similar distributions were observed when the population sizes were restricted to 100/population (inset, Fig. 1), or 25/population (data not shown). This analysis, together with the AMOVA results, illustrates that no genetic–geographic structuring is detected with this set of RM Y-STR haplotypes, since their high mutation rates have likely removed signals of shared population history, and has driven the high number of unique haplotypes. The low between-population differentiation and lack of substructure could be expected as a result of the high within-population diversities, and the effect of mutation rates on the estimation of θ [Meirmans and Hedrick, 2011]. As a practical consequence, no population substructure correction needs to be applied when using this RM Y-STR set in forensic or other applications, as is usually needed for other DNA marker systems. However, the lack of substructure correction required does not necessary infer a lack of structure in the distribution of haplotypes across geographic regions. Nonrandom distributions, caused by shared population histories and common origins, would infer the need to generate and utilize regional or meta-population-specific databases for frequency estimation. As such, the ability for RM Y-STRs to differentiate between male lineage within and between different populations and the relative distribution of haplotypes between regional groups was investigated.

### Value of RM Y-STRs in Improving Male Lineage Differentiation

When considering the utility of the RM Y-STR set for future applications in forensic, genealogical or anthropological genetic studies, it is informative to compare its properties to those of conventional Y-STRs, such as the 17 loci included in the Yfiler kit. Hence, RM YSTR and Yfiler haplotypes were compiled for 7,784 individuals across 65 populations as a subset of the global dataset presented here. In every diversity measure applied, the 13 RM YSTR set provided enhanced estimates relative to the 17-loci Yfiler set. Global haplotype diversity was increased from 0.99995 with Yfiler to 0.999997 with RM Y-STRs; the total number of haplotypes detected was increased from 6,975 to 7,714, and the number of singletons from 6,469 to 7,647. All regional groups showed more haplotypes and higher diversity estimates with RM Y-STRs relative to Yfiler, that is, haplotype diversity increased on average by 0.00226 and the number of singletons on average by 77 (largest increase of 1.56-fold in Aboriginal Australians) (Table 2). At the population level, there were increases in the number of haplotypes and in the haplotype diversity estimates for 56 (86.2%) of the 65 populations with the RM Y-STR set compared with Yfiler, whereas for six (9.2%) populations the same number of haplotypes were obtained because all males were already fully individualized with Yfiler (often in small sample-sized populations), and for three populations (4.6%), one haplotype fewer was detected with the RM Y-STRs than with Yfiler (Supp. Table S5).

**Table 2:** Comparison of RM Y-STR and Yfiler Haplotype Characteristics in a Global Set of 7,784 Individuals from 65 Populations Summarized for Regional Groups

Group	Individuals	Populations	Yfiler haplotypes	RM Y-STR haplotypes	Yfiler haplotype diversity	RM Y-STR haplotype diversity	Yfiler average percentage of differing loci	RM Y-STR average percentage of differing loci
North Africa	193	2	173	189	0.99792	0.99973	59%	73%
Central Asia	83	1	67	77	0.99060	0.99824	63%	77%
South Asia	497	6	450	483	0.99951	0.99988	68%	83%
East Asia	633	5	580	630	0.99952	0.99999	64%	82%
South East Asia	200	2	175	198	0.99759	0.99990	66%	81%
Aboriginal Australian	100	1	74	96	0.99152	0.99919	68%	80%
Native American	279	8	233	275	0.99761	0.99988	61%	82%
Admixed Native American	458	6	444	454	0.99986	0.99996	67%	81%
European	4,041	25	3,696	4,025	0.99991	0.999998	66%	82%
Migrant Sub-Saharan Africa	442	3	407	439	0.99909	0.99997	63%	81%
Migrant European	552	3	541	551	0.99993	0.99999	63%	81%
Migrant Asian	205	2	176	203	0.99737	0.99990	66%	83%
Biaka Pygmies	101	1	83	94	0.99505	0.99822	65%	83%
Global	7,784	65	6,975	7,714	0.99995	0.999997	65%	81%

**Figure 2:**
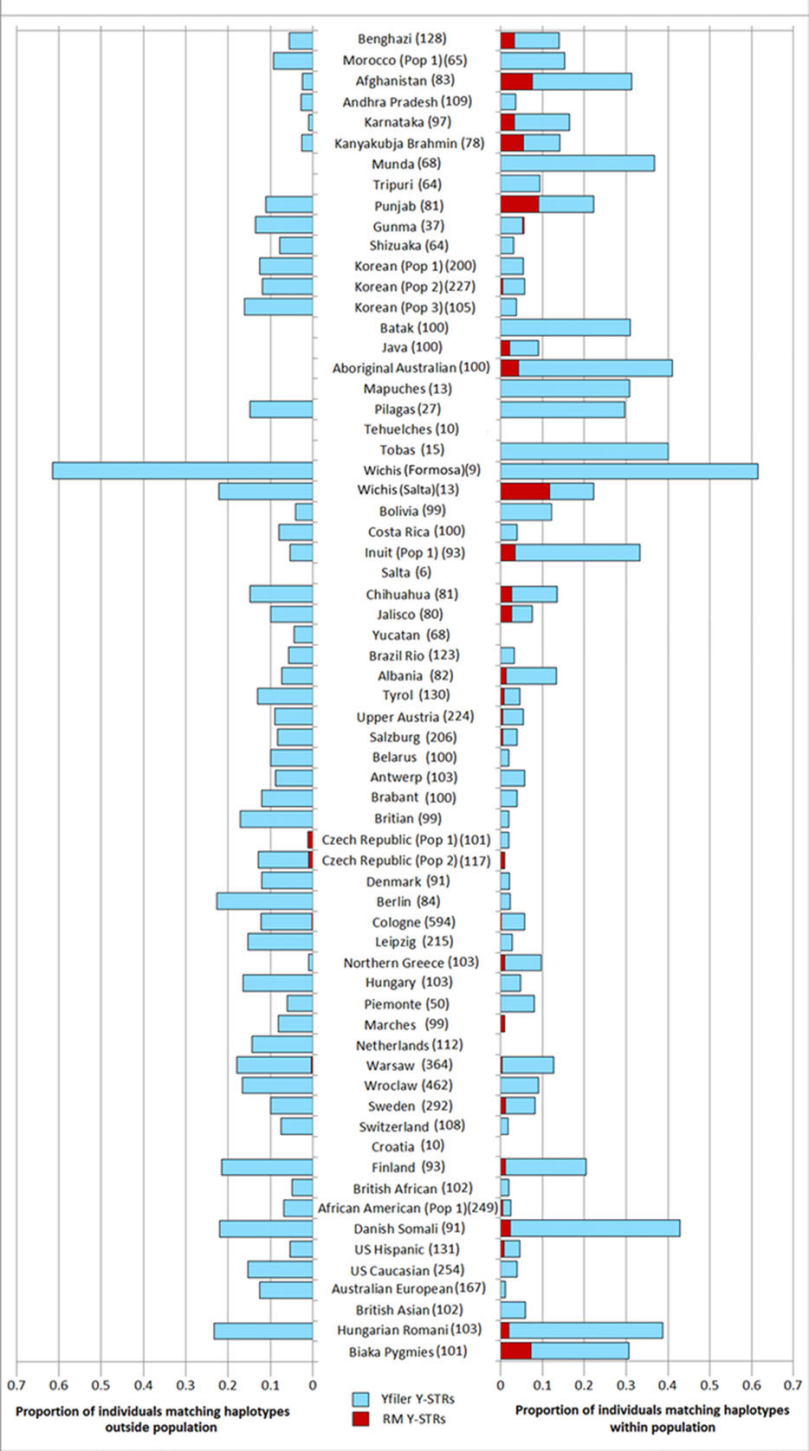
Proportion of individuals with haplotypes shared within populations (right) and between populations (left) for Yfiler (light blue bars) and the RM Y-STR set (dark red bars).

There was a significant difference between the RM Y-STR and the Yfiler sets in the level of haplotype sharing between individuals within regional groups, between populations, and within populations (Fig. 2). At the global level, 506 Yfiler haplotypes were shared between 1,318 individuals, compared with only 66 RM Y-STR haplotypes shared between 70 individuals. For Yfiler at the population level, only three (4.6%) of the 65 populations did not display any matching individuals either within or outside populations; 58 (89.2%) and 52 (80.0%), respectively, showed within and outside population haplotype matches. Within- and outside-population haplotype matches for Yfiler were as high as 62%, for the Wichis—a Native American group from Argentina (although sample size was *N* = 13). Notably, the level of outside-population matches compared with within-population matches was markedly higher for most populations for Yfiler haplotypes, whereas strikingly reduced for RM Y-STRs (Fig. 2). Of the 65 populations, 35 (53.8%) did not show any haplotype matches within and outside populations. Only 29 (44.6%) of the 65 populations displayed haplotype matches within populations, with the highest proportion (11%) observed in Argentinian Wichi from the Salta province. For 49 of the 58 populations that showed within-population Yfiler haplotype matches, no RM Y-STR haplotype matches were observed. The reduction in haplotype matches for RM Y-STRs relative to Yfiler is even more striking when considering outside-population matches. Only four (6.2%) populations displayed outside-population RM Y-STR matches, namely, two Czech population samples (one haplotype), and Cologne and Warsaw (one haplotype). The finding that 61 populations (93.8%) showed no outside population haplotype matches for RM Y-STRs contrasts strongly with those found with Yfiler, for which the corresponding number is 13 (20%).

**Figure 3:**
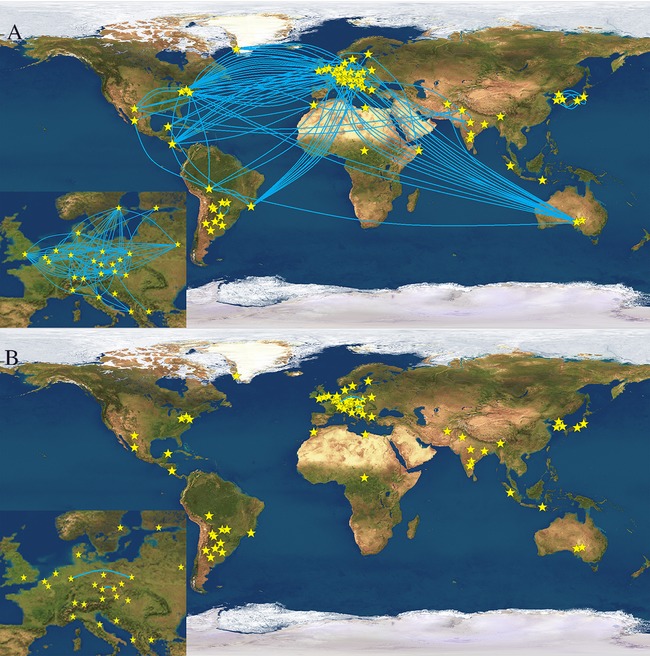
Geographic representation of pairwise between-population haplotype sharing. Blue lines connect population pairs showing shared haplotypes for **A**: Yfiler, and **B**: the RM Y-STRs set. Smaller insets show Europe enlarged.

Figure 3 provides an overview of the population pairs that showed between population haplotype matches for Yfiler (Fig. 3A, blue lines) and RM Y-STRs (Fig. 3B, blue lines). The significant reduction in between-population haplotype sharing (*t*_84_ = 8.091, *P* = 2.23 × 10^−11^) demonstrates the power of the RM Y-STR panel in male lineage differentiation. For RM Y-STRs, no haplotype sharing was observed between different populations sampled from the same countries, such as the five Indian, two Japanese, three Korean, three Austrian, two Belgian, three German, two Italian, two Polish, three US American, and two Hungarian populations, except for the two Czech populations. Further, almost no RM Y-STR haplotype sharing was observed between populations from the same geographic region such as the densely sampled continent of Europe except for Cologne and Warsaw. In contrast, many Yfiler haplotypes were shared between populations within countries, and between populations within regions such as Europe (see Fig. 3, insets).

**Figure 4:**
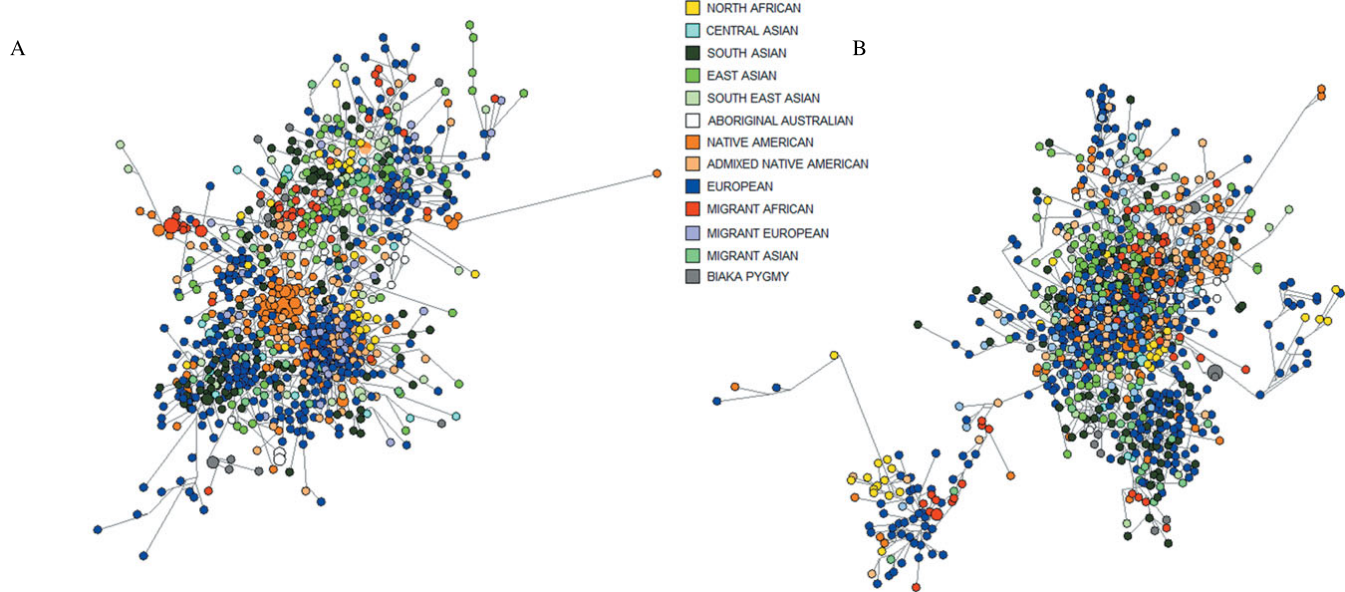
Weighted median-joining networks from single-copy Y-STRs for 1,000 individuals randomly selected from the total dataset, with regional geographic groups indicated by colors (see inset legend). **A**: Based on 15-loci Yfiler haplotypes (excluding DYS385a/b). **B**: Based on 10-loci RM Y-STR haplotypes (excluding DYF399S1, DYF403S1, and DYF404S1).

The lack of RM Y-STR haplotype sharing between populations is not limited to the full 13-loci haplotype. Network analysis of 1,000 individuals, selected at random in the same population proportions as the full set of 7,784 samples and based on single-copy Y-STRs, displayed little geographic clustering with RM Y-STR haplotypes (Fig. 4B, perhaps with the exception of North Africans), whereas with Yfiler haplotypes for the same individuals (Fig. 4A), geographic clustering is seen with several groups. For RM Y-STRs, the network was constructed based on nine single-copy Y-STRs (excluding DYF387S1, DYF399S1, DYF403S1, and DYF404S1), and for Yfiler, based on 15 Y-STRs (excluding DYS385a/b). Unlike the Yfiler network, the RM Y-STR network provides almost no clustering of haplotypes according to geographic regions of sampling (except some of the North African haplotypes), which underlines the lack of population substructure as also seen in the MDS and AMOVA of the complete data. Hence, for RM Y-STRs, the need for regional (metapopulation) reference databases for haplotype frequency estimation in forensic and other applications is strongly reduced compared with conventional Y-STRs such as those in the Yfiler kit. Combining the RM Y-STR set and the Yfiler set to generate 30-marker Y-STR haplotypes resulted in the individualization of 25 additional men of the 137 not already individualized with the RM Y-STR set alone. While this does represent a slight improvement on the global scale, from 7,714 to 7,737 haplotypes, it is clear that the vast majority of differentiation was achieved by the RM Y-STR set alone.

### Ability of RM Y-STRs to Detect Hidden Paternal Relationships

The observation of RM Y-STR haplotype sharing within, but (almost) not between, populations might indicate that RM Y-STRs allow the detection of unknown (i.e., hidden) paternal relationships in populations. If true, more shared RM Y-STR haplotypes would be expected between putatively unrelated individuals sampled from rural areas, where male relatives often stay in the region (and thus can be sampled), and especially from endogamous populations, than among putatively unrelated individuals sampled from urban areas, where male relatives tend to not to stay (and thus cannot be sampled). To test this hypothesis, all populations were designated according to their urbanization status—each set of males was classified as either urban (genetically moderately outbred), rural (more likely to be genetically inbred), mixed (males from both rural and urban populations, in unspecified proportions), or endogamous (populations subscribing to the cultural practice of endogamy—restricting marriage outside of an individual's social class, ethnic group, or tribe). In support of the RM Y-STR set's ability to detect hidden paternal relationships, we indeed see significantly lower haplotype diversity and lower proportions of unique haplotypes in the endogamous group than in the rural group than in the urban group (diversity *t*_2_ = 7,960, *P* = 1.6 × 10^−8^, shared haplotypes *t*_2_ = 1.347, *P* = 0.310) (Table 3). Although the sample sizes differ considerably between the three categories, this does not affect the conclusions as simulations performed with equalized sample numbers (*n* = 558 per category) showed that the RM Y-STRs still result in decreased diversity in endogamous and rural populations compared with urban populations (data not shown; diversity *t*_2_ = 8936, *P* = 1.3 × 10^−8^, shared haplotypes *t* = 1.117, *P* = 0.380).

**Table 3:** RM Y-STR Haplotype Characteristics in a Global Set of 12,272 Individuals from 111 Populations Summarized for Groups Based on Urbanization Status

Population classification	Populations	Individuals	Haplotypes	Singletons	Haplotype diversity	Proportion of nonunique haplotypes
Endogamous	7	558	512	478	0.999614	0.143
Rural	20	1,369	1,354	1,340	0.999982	0.021
Urban	51	7,198	7,164	7,131	0.999999	0.009
Mixed	26	2,379	2,361	2,342	0.999994	0.015
Unknown	7	770	767	764	0.999990	0.008

On the other hand, these data also demonstrate that even in populations with considerable hidden paternal relationships, such as in endogamous populations, the RM Y-STR set still allows the differentiation of a large number of men. A comparison between Yfiler and the RM Y-STR set for the available populations (Table 4) illustrates the improved value of RM Y-STRs relative to Yfiler in differentiating males when grouping populations into urban, rural, and endogamous. Most tellingly, even in the endogamous group, the haplotype diversity increased and the proportion of shared (i.e., nonunique) haplotypes decreased from 0.99947 and 0.101 with Yfiler to 0.99978 and 0.045 with the RM Y-STR set, respectively, in the rural group from 0.99916 and 0.157 to 0.99997 and 0.008, and even in the urban from 0.99994 and 0.08 to 0.999998 and 0.005.

**Table 4:** Comparison of RM Y-STRs and Yfiler Haplotype Characteristics in a Global Set of 7,784 Individuals from 65 Populations Summarized for Groups Based on Urbanization Status

Population classification	*N*	Yfiler ht	RM Y-STR ht	Yfiler singletons	RM Y-STR singletons	Yfiler haplotype diversity	RM Y-STR haplotype diversity	Yfiler proportion of nonunique haplotypes	RM Y-STR proportion of nonunique haplotypes	Yfiler percentage average allelic difference	RM Y-STR percentage average allelic difference
Endogamous	466	419	445	380	427	0.99947	0.99978	0.101	0.045	68%	83%
Rural	714	602	708	537	703	0.99916	0.99997	0.157	0.008	70%	85%
Urban	4,974	4,577	4,951	4,318	4,929	0.99994	0.999998	0.080	0.005	68%	83%
Mixed	1,422	1,332	1,407	1,267	1,392	0.99988	0.99999	0.063	0.011	67%	84%
Unknown	208	181	205	169	202	0.99684	0.99986	0.130	0.014	64%	83%

### Value of RM Y-STRs for Male Relative Differentiation

In addition, we tested the value of RM Y-STRs for differentiating male relatives. On a theoretical level, the rate of relative differentiation per meiosis can be calculated using the mutation rates of each locus within a genotyping panel (see *Materials and Methods* section). For the Yfiler set, it is estimated that a mutation at one or more of the 17 Y-STR loci will occur with a probability of 0.047 (95% confidence interval 0.038–0.057) per meiosis, which for the RM Y-STR set is more than fourfold higher at 0.195 (95% CI 0.177–0.21). Notably, for the recently released PowerPlex Y-23 kit (Promega), which targets all 17 Yfiler markers together with two of the 13 RM Y-STR markers and four additional Y-STRs, this value is 0.092 (95% CI 0.077–0.107), nearly twofold higher than for Yfiler, but more than twofold lower than for the RM Y-STR set studied here.

**Figure 5:**
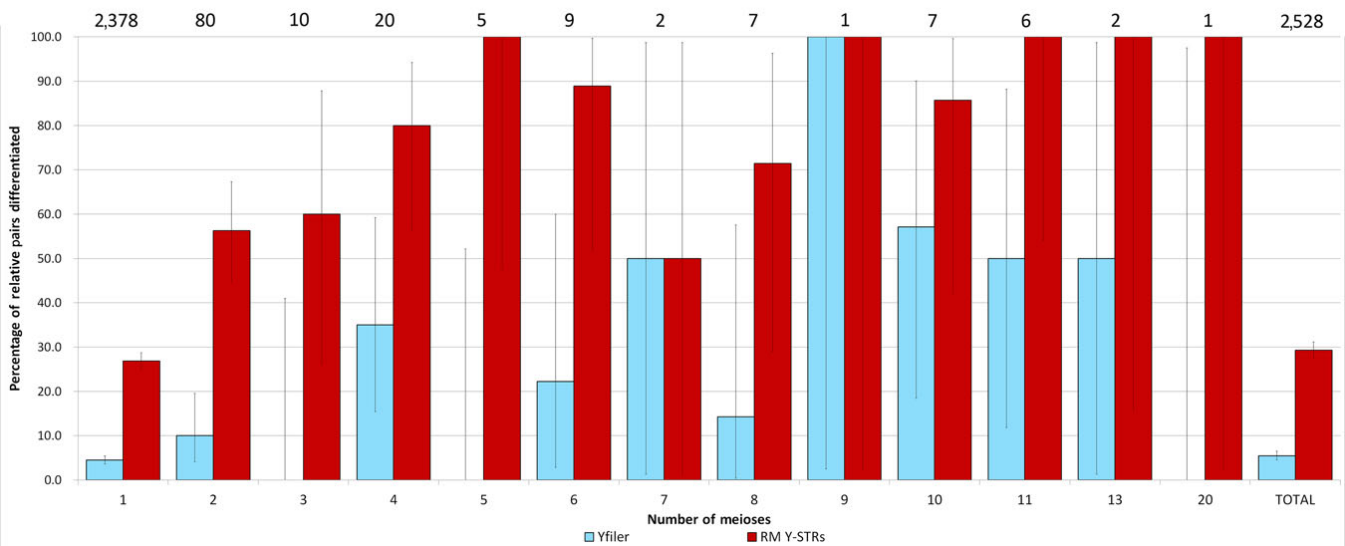
Empirical male relative differentiation using 2,528 paternal relative pairs separated by one to 20 meioses for Yfiler (light blue bars) and the RM Y-STR set (dark red bars). The data combine the new 2,372 relative pairs from the current study with the previously used 156 relative pairs [Ballantyne et al., 2012]. Values above the bars indicate the absolute number of relative pairs the estimated differentiation rate is based upon. Error bars represent binomial confidence intervals.

To compare the theoretical expectation for the RM Y-STR and the Yfiler sets with empirical data, we genotyped 2,372 pairs of male relatives previously confirmed by autosomal DNA analysis (2,339 father–son pairs, 30 brother pairs, and three uncle–nephew pairs), confirmed the observed allelic differences by independent genotyping (and some by sequencing), and combined these new data with those from the 156 relative pairs separated by one to 20 paternal genetic transfers (or meioses) described previously [Ballantyne et al., 2012]. In this combined dataset, the RM Y-STR set allows differentiation by at least one allelic difference (i.e., mutation) in at least one locus in 742 (29%) of the total 2,528 pairs related by one to 20 generations, whereas Yfiler only allowed the separation of 118 (5.5%) of a subset of 2,161 of these relative pairs (Fig. 5). In particular, the RM Y-STR set differentiated fathers from their sons in 26.9% of the cases versus 4.5% with Yfiler, and brothers from each other in 56.3% of the cases versus 10.0% with Yfiler.

It should be noted, however, that only for the 2,378 father–son pairs investigated here, was the sample size large enough to allow reliable conclusions, whereas for more distantly related males, future studies need to deliver more data. However, as the chance of mutations increases with each meiosis, it is expected that the more distantly related men are, the higher the chance that they will have different RM Y-STR haplotype; a trend that can be seen in our data (Fig. 5). The importance of sufficient sample size is illustrated in the following for father–son pairs. Our initial estimate of father–son differentiation was 70% based on only 20 pairs [Ballantyne et al., 2010], which was subsequently revised to 49% based on 39 pairs, including the initial 20 pairs [Ballantyne et al., 2012]. In the present study, we managed to increase the number of father–son pairs drastically to 2,378 pairs, now achieving a differentiation rate of 26.9% (95% confidence interval 25.1%–28.7%). Notably, the empirical rate achieved, now based on a large sample size, corresponds more closely to the theoretical estimate of 19.5%.

Removing DYF403S1a+b from the RM Y-STR panel resulted in the nondifferentiation of 84 relative pairs that could be differentiated when DYF403S1a+b was included; the overall relative differentiation rate dropped to 26%. However, the difference only occurred in pairs with fewer than five meioses separating the individuals—in all other pairs, there were sufficient mutations at other RM Y-STR loci to allow differentiation (data not shown).

As pointed out previously [Ballantyne et al. 2012], RM Y-STRs are not particularly useful for paternity testing or familial searching because of their high mutation rates and the resultant power of differentiation of male relatives. If, however, they are used for such purposes, correction factors would be needed to compensate for the likely observed mutations in the probability calculations. It has been suggested before that the presence of at least three mutations at any Y-STR loci (most commonly with the 17 Yfiler Y-STRs) is sufficient to exclude paternity [Kayser and Sajantila 2001; Gjertson et al., 2007]. In the current dataset however, 3.8% of the DNA-confirmed father–son pairs displayed three or four RM Y-STR mutations; as such, the number of mutations constituting exclusion should be increased when using the RM Y-STRs. As outlined elsewhere (Ballantyne et al. 2012), instead of an ad hoc cut-off, a dynamic threshold shall be used, estimated from the number of Y-STR loci genotyped and their locus-specific mutation rates. Because three mutations have also been observed occasionally among 17 Yfiler loci in DNA-confirmed father–son pairs [Goedbloed et al. 2009], this notion also applies to conventional Y-STR sets such as Yfiler, which due to their moderate mutation rates in principle are more appropriate for paternity and kinship testing than RM Y-STRs.

## Conclusions

The large worldwide dataset compiled, presented, and analyzed here demonstrates the exceptional value of the RM Y-STR panel for differentiating male lineages on a global and regional scale. Many of the current limitations of Y-chromosome applications are reduced with this RM Y-STR set, providing increased utility and effectiveness to the genetic analysis of male populations and lineages. In particular, this 13 RM Y-STR set provides near-complete paternal lineage differentiation in general populations as well as in populations with otherwise reduced Y-chromosome diversity, due to peculiarities in population history or cultural practices. The effects of this near-complete male individualization will be of great benefit to numerous fields using the Y-chromosome genetics to investigate male lineages, such as in genealogical studies (e.g., to detect extrapair paternity or adventitious haplotype matches within different surnames/lineages), population genetic and genetic history studies (e.g., to assist in differentiating between lineages shared between populations with common history), and in forensic applications (e.g., to reduce the inclusion of innocent individuals in investigations due to adventitious haplotype matches). Moreover, the immense value of this RM Y-STR set to differentiate between both close and distant male relatives will have beneficial effects on these same fields, especially in forensic genetics, providing increased confidence that haplotype matches between unknown samples such as those from crime scenes and reference samples such as those from suspects are due to individual identity, rather than relatedness.

On the other hand, however, the extreme degree of RM Y-STR haplotype diversity highlights the limitations of the current approach for placing a statistical weight on Y-STR haplotype matches by using empirically derived haplotype frequency estimates obtained from Y-STR haplotype reference databases. Although already noted with the currently used Y-STR sets such as Yfiler, the problem that an observed haplotype is not present in the large and growing reference database, and how to deal with rare haplotypes in obtaining frequency estimates and estimating match probabilities for which no consensus has been reached yet [Krawczak, 2001; Brenner, 2010; Buckleton et al., 2011; Willuweit et al., 2011], will become drastically increased when using RM Y-STRs, as can be seen from the data presented here. Therefore, new statistical solutions shall be developed to estimate the weight of a RM Y-STR haplotype match with particular relevance for forensic applications.

To assist future studies utilizing RM Y-STRs, complete dataset of RM Y-STR and Yfiler haplotypes obtained from the 12,272 individuals analyzed here is made available via Supp. Table S6, with allele frequencies for individual populations, regional groups, and the complete dataset summarized in Supp. Table S7. Relative pair haplotypes for RM Y-STRs and Yfiler are available in Supp. Table S8.

## Supporting Information

Disclaimer: Supplementary materials have been peer-reviewed but not copyedited.

**Table S1.** RM Y-STR Primer sequences and concentrations used in three multiplex PCRs **Table S2.** Reaction composition and conditions for the three RM Y-STR multiplexes used **Table S3.** RM Y-STR repeat structure, allelic designations and control 007 genotype **Table S4.** RM Y-STR haplotype characteristics in a global set of 12,272 individuals from 111 populations summarized for population samples ordered according to regional groups **Table S5.** Comparison of Yfiler and RM Y-STR haplotype characteristics in a global set of 7,784 individuals from 65 populations summarized for population samples ordered according to regional groups

**Table S6.** RM Y-STR and Yfiler haplotypes obtained from the 12,272 individuals in 111 global populations

**Table S7.** Allele frequencies and ranges for individual populations, regional groups, and the complete dataset for each RM Y-STR marker

**Table S8.** RM Y-STR and Yfiler haplotypes for male relative pairs
