# Supplementary material for: Toward Male Individualization with Rapidly Mutating Y-Chromosomal Short Tandem Repeats
Source: Hum Mutat. 2014 Jun 10;35(8):1021–32. doi: 10.1002/humu.22599 (PMC4145662; doi:10.1002/humu.22599)
Supplement: Supplementary file 1 [file humu0035-1021-SD1.pdf]

Supp. Table S1. RM Y-STR Primer sequences and concentrations used in three multiplex PCRs

Multiplex	Primers	Sequence 5´- 3´	Concentration
**RM 1**	DYS576 F	6-FAM-TTGGGCTGAGGAGTTCAATC	0.1 µM
	DYS576 R	GGCAGTCTCATTTCCTGGAG	0.1 µM
	DYS570 F	TAMRA-GAACTGTCTACAATGGCTCACG	0.3 µM
	DYS570 R	TCAGCATAGTCAAGAAACCAGACA	0.3 µM
	DYF387S1 F	JOE-GCCTGGGTGACAGAGCTAGA	0.2 µM
	DYF387S1 R	GCCACAGTGTGAGAAGTGTGA	0.2 µM
	DYF399S1 F	6-FAM-GGGTTTTCACCAGTTTGCAT	0.4 µM
	DYF399S1 R	CCATGTTTTGGGACATTCCT	0.4 µM
**RM 2**	DYS626 F	6-FAM-GCAAGACCCCATAGCAAAAG	0.2 µM
	DYS626 R	AAGAAGAATTTTGGGACATGTTT	0.2 µM
	DYS627 F	JOE-CTAGGTGACAGCGCAGGATT	0.2 µM
	DYS627 R	GGATAATGAGCAAATGGCAAG	0.2 µM
	DYS526 F	6-FAM-TCTGGTGAACTGATCCAAACC	0.2 µM
	DYS526 R	GGGTTACTTCGCCAGAAGGT	0.2 µM
	DYS518 F	TAMRA-GGCAACACAAGTGAAACTGC	0.2 µM
	DYS518 R	TCAGCTCTTACCATGGGTGAT	0.2 µM
**RM 3**	DYS612 F	6-FAM-CCCCATGCCAGTAAGAATA	0.15 µM
	DYS612 R	GTGAGGGAAGGCAAAAGAAAA	0.15 µM
	DYF404S1 F	JOE-GGCTTAAGAAATTTCAACGCATA	0.15 µM
	DYF404S1 R	CCATGATGGAACAATTGCAG	0.15 µM
	DYS449 F	6-FAM-TGAATATTTTCCCTTAACTTGTGTGA	0.075 µM
	DYS449 R	CCATTGCACTCTAGGTTGGAC	0.075 µM
	DYS547 F	6-FAM-TCCATGTTACTGCAAAATACAC	0.3 µM
	DYS547 R	TGACAGAGCATAAACGTGTC	0.3 µM
	DYF403S1 F	JOE-CAAAATTCATGTGGATAATGA	0.5 µM
	DYF403S1 R	ACAGAGCAGGATTCCATCTA	0.5 µM

Supp. Table S2. Reaction composition and conditions for the three RM Y-STR multiplexes used

<table>
<tr><th>Multiplex</th><th>Multiplex Component</th><th>Volume (µl per 10µl reaction)</th><th>Cycling parameters</th></tr>
<tr><td rowspan="7">RM 1</td><td>Qiagen Multiplex PCR Master Mix</td><td>5</td><td rowspan="22">94°C
10min

94°C 30sec
65°C -1°C/cycle 30sec
10x
72°C 60sec

94°C 30sec
50°C 30sec
25x
72°C 60sec

60°C
45min
15°C ∞</td></tr>
<tr><td>DYS576 F+R</td><td>0.1</td></tr>
<tr><td>DYF387S1 F+R</td><td>0.2</td></tr>
<tr><td>DYS570 F+R</td><td>0.3</td></tr>
<tr><td>DYF399S1 F+R</td><td>0.4</td></tr>
<tr><td>Water</td><td>3</td></tr>
<tr><td>Template</td><td>1</td></tr>
<tr><td rowspan="7">RM 2</td><td>Qiagen Multiplex PCR Master Mix</td><td>5</td></tr>
<tr><td>DYS526 F+R</td><td>0.2</td></tr>
<tr><td>DYS626 F+R</td><td>0.2</td></tr>
<tr><td>DYS627 F+R</td><td>0.2</td></tr>
<tr><td>DYS518 F+R</td><td>0.2</td></tr>
<tr><td>Water</td><td>3.2</td></tr>
<tr><td>Template</td><td>1</td></tr>
<tr><td rowspan="8">RM 3</td><td>Qiagen Multiplex PCR Master Mix</td><td>5</td></tr>
<tr><td>DYS612 F+R</td><td>0.15</td></tr>
<tr><td>DYF404S1 F+R</td><td>0.15</td></tr>
<tr><td>DYS449 F+R</td><td>0.075</td></tr>
<tr><td>DYS547 F+R</td><td>0.3</td></tr>
<tr><td>DYF403S1 F+R</td><td>0.5</td></tr>
<tr><td>Water</td><td>2.825</td></tr>
<tr><td>Template</td><td>1</td></tr>
</table>

Supp. Table S3. RM Y-STR repeat structure, allelic designations and control 007 genotype

RM Y-STR	Repeat Structure	Allele Designation	Control DNA 007 genotype	Allele Range Observed	Mutation Rate*
**DYF387S1**	(AAAG)$_3$(GTAG)$_1$(GAAG)$_4$N$_{20}$(GAAG)n(AAAG)o	13+n+o	**35, 37**	**28-45.2**	1.59x10^{-2}
**DYS399S1**	(GAAA)$_3$N$_{7-8}$(GAAA)n	5+n	**24, 26.1**	**14-31.3**	7.73x10^{-2}
**DYF403S1a**	(TTCT)nNo(TTCT)p	n+o+p	**11, 13, 18**	**5-24**	3.10x10^{-2}
**DYF403S1b**	(TTCT)n**N$_2$**(TTCT)o(TTCC)p(TTCT)q	n+o+p+q	**51**	**38-62**	1.19x10^{-2}
**DYF404S1**	(TTTC)n	n	**14, 16**	**7-21**	1.25x10^{-2}
**DYS449**	(TTTC)n**N$_{50}$**(TTTC)o	n+o	**30**	**23-40**	1.22x10^{-2}
**DYS518**	(AAAG)$_3$(GAAG)$_1$(AAAG)n(GGAG)$_1$(AAAG)$_4$**N$_6$**(AAAG)o	9+n+o	**37**	**32-52.2**	1.84x10^{-2}
**DYS526a**	(CCTT)n	n	**14**	**7-21**	2.72x10^{-3}
**DYS526b**	(CTTT)o(CCTT)p**N$_{113}$**(CCTT)n	n+o+p	**36**	**29-44**	1.25x10^{-2}
**DYS547**	(CCTT)n**T**(CTTC)o**N$_{56}$**(TTTC)p**N$_{10}$**(CCTT)$_4$(TCTC)$_1$(TTTC)q	n+o+p+5+q	**49**	**39-57**	2.36x10^{-2}
**DYS570**	(TTTC)n	n	**17**	**10-24**	1.24x10^{-2}
**DYS576**	(AAAG)n	n	**19**	**11-23**	1.43x10^{-2}
**DYS612**	(CCT)$_5$(CTT)$_1$(TCT)$_4$(CCT)$_1$(TCT)n	11+n	**37**	**25-43**	1.45x10^{-2}
**DYS626**	(GAAA)n**N$_{24}$(GAAA)$_3$N$_6$**(GAAA)$_5$(AAA)$_1$(GAAA)o(GAAG)$_1$(GAAA)$_3$	n+10+o	**30**	**22-52**	1.22x10^{-2}
**DYS627**	(AGAG)$_3$(AAAG)n	3+n	**21**	**12-27**	1.23x10^{-2}

Segments in **bold** are not counted in the allele designation.

* Mutation rates from Ballantyne et al., 2010

Supp. Table S4. RM Y-STR haplotype characteristics in a global set of 12,272 individuals from 111 populations summarized for population samples ordered according to regional groups

Group	Population	Number individuals	Number haplotypes	Haplotype diversity	θ	Average number of locus differences
Sub-Saharan Africa	Bakangos (Angola)	25	25	1.0	*0*	16.07
	Kimbundos (Angola)	26	25	0.99692	0.00264550	15.76
	Ovimbundos (Angola)	44	44	1.0	*0*	15.79
	Guinea-Bissau	92	91	0.99976	0.00022878	16.99
	Xhosa (South Africa)	116	115	0.99985	0.00014486	15.50
North Africa	Benghazi (Libya)	128	124	0.99938	0.00086889	15.47
	Tripoli (Libya)	105	104	0.99982	0.00017634	16.29
	Morocco (Population 1)	151	149	0.99982	0.00016752	15.45
	Morocco (Population 2)	68	68	1.0	*0*	15.12
Middle East	Turkey	100	100	1.0	*0*	15.26
Central Asia	Afghanistan	86	80	0.99836	0.00124160	16.18
South Asia	Andhra Pradesh (India)	109	109	1.0	*0*	17.00
	Karnataka (India)	97	94	0.99936	0.00056963	16.95
	Kanyakubja Brahmin (India)	78	74	0.99867	0.00108563	16.83
	Munda (India)	68	68	1.0	*0*	16.23
	Tripuri (India)	64	64	1.0	*0*	16.44
	Punjab (Pakistan)	95	85	0.99754	0.00208782	17.17
	Brahui (Pakistan)	77	77	1.0	*0*	16.86
	Pathan (Pakistan)	73	73	1.0	*0*	16.44
East Asia	Kanto (Japan)	147	147	1.0	*0*	16.79
	Gunma (Japan)	37	35	0.99700	0.00242433	16.63
	Shizuaka (Japan)	64	64	1.0	*0*	17.07

Group	Population	Number individuals	Number haplotypes	Haplotype diversity	θ	Average number of locus differences
	Korean (Population 1)	200	200	1.0	*0*	17.20
	Korean (Population 2)	227	226	0.99996	0.00003831	17.22
	Korean (Population 3)	182	182	1.0	*0*	17.29
	Bai (China)	110	110	1.0	*0*	16.65
South East Asia	Thai (Thailand)	120	120	1.0	*0*	17.40
	East Timor	110	108	0.99967	0.00031030	17.34
	Batak (Indonesia)	104	104	1.0	*0*	16.87
	Java (Indonesia)	100	98	0.99960	0.00037311	16.38
	Filipino (Philippines)	97	97	1.0	*0*	14.93
	Malay (Singapore)	103	103	1.0	*0*	17.37
Aboriginal Australian		100	96	0.99919	0.00068892	16.8
Native American	Tehuelches (Argentina)	10	10	1.0	*0*	16.16
	Bolivia	99	99	1.0	*0*	16.78
	Mocovies (Argentina)	7	7	1.0	*0*	15.38
	Pilagas (Argentina)	31	31	1.0	*0*	14.91
	Tobas (Argentina)	15	15	1.0	*0*	14.62
	Mapuches (Argentina)	13	13	1.0	*0*	16.58
	Wichis (Salta Province, Argentina)	9	8	0.97222	0.01818182	15.08
	Wichis (Formosa Province, Argentina)	14	14	1.0	*0*	11.38
	Inuit (Greenland, Population 1)	95	92	0.99910	0.00137764	16.90
	Inuit (Greenland, Population 2)	72	69	0.99883	0.00099431	17.01
Admixed Native American	Chubut (Argentina)	50	50	1.0	*0*	16.42
	Corrientes (Argentina)	25	25	1.0	*0*	16.45

**Group**	**Population**	**Number individuals**	**Number haplotypes**	**Haplotype diversity**	***θ***	**Average number of locus differences**
	Formosa (Argentina)	25	25	1.0	*0*	16.16
	Misiones (Argentina)	25	25	1.0	*0*	17.15
	Rio Negro (Argentina)	50	50	1.0	*0*	16.69
	Salta (Argentina)	25	25	1.0	*0*	16.59
	Rio de Janeiro (Brazil)	136	136	1.0	*0*	19.12
	Costa Rica	102	102	1.0	*0*	17.16
	Buenos Aires (Argentina)	97	96	0.99979	0.00020614	17.15
	Chihuahua (Mexico)	81	79	0.99938	0.00055953	16.97
	Jalisco (Mexico)	80	78	0.99937	0.00057300	17.14
	Yucatan (Mexico)	68	68	1.0	*0*	17.41
European	Albania	82	81	0.99970	0.00028686	17.00
	Tyrol (Austria)	130	129	0.99988	0.00011566	17.47
	Upper Austria	226	225	0.99996	0.00003864	17.51
	Salzburg (Austria)	206	205	0.99995	0.00004645	17.59
	Belarus	100	100	1.0	*0*	17.15
	Antwerp (Belgium)	103	103	1.0	*0*	16.90
	Brabant (Belgium)	100	100	1.0	*0*	17.01
	Bosnia	100	100	1.0	*0*	16.12
	Britain	99	99	1.0	*0*	16.77
	Croatia	112	111	0.99984	0.00015526	15.41
	Czech Republic (Population 1)	102	102	1.0	*0*	17.12
	Czech Republic (Population 2)	133	132	0.99989	0.00011056	17.56
	Denmark	92	92	1.0	*0*	16.81
	Estonia	109	109	1.0	*0*	16.66
	Finland (Population 1)	103	103	1.0	*0*	14.28

**Group**	**Population**	**Number individuals**	**Number haplotypes**	**Haplotype diversity**	***θ***	**Average number of locus differences**
	Finland (Population 2)	96	95	0.99978	0.00021039	16.47
	Berlin (Germany)	208	207	0.99995	0.00004557	16.52
	Cologne (Germany, Population 1)	97	97	1.0	*0*	17.37
	Cologne (Germany, Population 2)	599	598	0.999994	0.00000555	16.26
	Leipzig (Germany)	219	219	1.0	*0*	14.16
	Northern Greece	108	107	0.99983	0.00016681	17.55
	Hungary	103	103	1.0	*0*	17.38
	Piemonte (Italy)	50	50	1.0	*0*	16.79
	Sicily (Italy)	50	50	1.0	*0*	17.34
	Marches (Italy)	100	99	0.99980	0.00019414	17.06
	Lithuania	84	84	1.0	*0*	16.78
	Netherlands	112	112	1.0	*0*	16.45
	Norway	152	152	1.0	*0*	16.81
	Northern Poland	100	100	1.0	*0*	17.03
	Warsaw (Poland)	369	368	0.999985	0.00001457	16.57
	Wroclaw (Poland)	467	467	1.0	*0*	16.51
	Latvia	132	132	1.0	*0*	16.97
	Slovakia	100	100	1.0	*0*	17.03
	Slovenia	128	126	0.99975	0.00023121	17.06
	Sweden	292	289	0.99993	0.00006777	17.02
	Switzerland	108	108	1.0	*0*	16.99
	Kiev (Ukraine)	103	103	1.0	*0*	16.85
	Galicia (Spain)	44	44	1.0	*0*	17.14
Migrant Sub-Saharan Africa	African American (Population 1)	100	100	1.0	*0*	16.71
	Reunion African	57	56	0.99937	0.00058445	14.94

Group	Population	Number individuals	Number haplotypes	Haplotype diversity	*θ*	Average number of locus differences
	British African	102	102	1.0	*0*	16.67
	African American (Population 2)	312	311	0.99998	0.00002035	17.06
	Danish Somali	92	90	0.99952	0.00043818	14.70
Migrant Asian	Reunion Indian	58	58	1.0	*0*	14.76
	Reunion Creole	92	90	0.99952	0.00043818	14.05
	Singapore Indian	102	102	1.0	*0*	17.29
	British Asian	102	102	1.0	*0*	17.67
	US Asian	88	88	1.0	*0*	17.49
	Singapore Han Chinese	104	104	1.0	*0*	17.40
	Hungarian Romani	103	101	0.99962	0.00035240	16.81
Migrant European	US Hispanic	219	218	0.99996	0.00004114	17.25
	US European	345	345	1.0	*0*	16.83
	Australian European	167	167	1.0	*0*	16.96
Bhutan	Monpa	40	31	0.98590	0.00918663	12.92
	Lhokpu	38	26	0.97724	0.02064797	7.72
Biaka Pygmy		101	94	0.99822	0.00225545	17.50

Supp. Table S5. Comparison of Yfiler and RM Y-STR haplotype characteristics in a global set of 7,784 individuals from 65 populations summarized for population samples ordered according to regional groups

Group	Population	Number individuals	Number Yfiler haplotypes	Number RM Y-STR haplotypes	Yfiler haplotype diversity	RM Y-STR haplotype diversity	Yfiler Average % of differing loci	RM Y-STR Average % of differing loci
North Africa	Benghazi (Libya)	128	116	124	0.997047	0.999385	58%	74%
	Morocco (Population 2)	65	59	65	0.996394	1.0	55%	72%
Middle East	Afghanistan	83	67	77	0.992507	0.998237	63%	77%
South Asia	Andhra Pradesh (India)	109	107	109	0.99966	1.0	66%	81%
	Karnataka (India)	97	89	94	0.998282	0.999356	67%	81%
	Kanyakubja Brahmin (India)	78	72	74	0.997669	0.998668	63%	80%
	Munda (India)	68	53	68	0.988586	1.0	49%	77%
	Tripuri (India)	64	61	64	0.998512	1.0	61%	78%
	Punjab (Pakistan)	81	71	74	0.997068	0.997531	65%	82%
East Asia	Gunma (Japan)	37	36	35	0.998498	0.996997	59%	79%
	Shizuaka (Japan)	64	63	64	0.999504	1.0	61%	81%
	Korean (Population 1)	200	194	200	0.999648	1.0	65%	82%
	Korean (Population 2)	227	219	226	0.999532	0.999961	60%	82%
	Korean (Population 3)	105	103	105	0.999634	1.0	65%	83%
South East Asia	Batak (Indonesia)	100	80	100	0.994343	1.0	64%	80%
	Java (Indonesia)	100	95	98	0.998081	0.999596	63%	78%

Group	Population	Number individuals	Number Yfiler haplotypes	Number RM Y-STR haplotypes	Yfiler haplotype diversity	RM Y-STR haplotype diversity	Yfiler Average % of differing loci	RM Y-STR Average % of differing loci
Aboriginal Australian		100	74	96	0.991515	0.999192	68%	80%
Native American	Tehuelches (Argentina)	10	10	10	1	1.0	60%	77%
	Bolivia	99	93	99	0.998763	1.0	56%	80%
	Pilagas (Argentina)	27	23	27	0.981481	1.0	45%	71%
	Tobas (Argentina)	15	12	15	0.971429	1.0	36%	70%
	Mapuches (Argentina)	13	11	13	0.974359	1.0	51%	79%
	Wichis (Salta Province, Argentina)	9	8	8	0.972222	0.972222	40%	72%
	Wichis (Formosa Province, Argentina)	13	7	13	0.794872	1.0	20%	54%
	Inuit (Greenland, Population 1)	93	72	90	0.990182	0.999065	66%	80%
Admixed Native American	Salta (Argentina)	6	6	6	1	1.0	69%	82%
	Rio de Janeiro (Brazil)	123	121	123	0.999733	1.0	65%	76%
	Costa Rica	100	98	100	0.999596	1.0	67%	82%
	Chihuahua (Mexico)	81	75	79	0.99784	0.999383	66%	81%
	Jalisco (Mexico)	80	77	78	0.999051	0.999367	66%	82%
	Yucatan (Mexico)	68	68	68	1	1.0	68%	83%

Group	Population	Number individuals	Number Yfiler haplotypes	Number RM Y-STR haplotypes	Yfiler haplotype diversity	RM Y-STR haplotype diversity	Yfiler Average % of differing loci	RM Y-STR Average % of differing loci
European	Albania	82	76	81	0.997892	0.999699	60%	81%
	Tyrol (Austria)	130	127	129	0.999642	0.999881	66%	83%
	Upper Austria	224	218	223	0.99976	0.99996	67%	83%
	Salzburg (Austria)	206	202	205	0.999811	0.999953	67%	84%
	Belarus	100	99	100	0.999798	1.0	60%	82%
	Antwerp (Belgium)	103	100	103	0.999429	1.0	62%	80%
	Brabant (Belgium)	100	98	100	0.999596	1.0	61%	81%
	Britain	99	98	99	0.999794	1.0	59%	80%
	Croatia	10	10	10	1	1.0	43%	65%
	Czech Republic (Population 1)	101	101	101	0.999703	1.0	62%	82%
	Czech Republic (Population 2)	117	117	116	1	0.999853	64%	84%
	Denmark	91	90	91	0.999756	1.0	63%	80%
	Finland (Population 2)	93	81	92	0.995792	0.999766	64%	79%
	Berlin (Germany)	84	83	84	0.999713	1.0	64%	74%
	Cologne (Germany, Population 2)	594	576	593	0.999881	0.999994	65%	77%
	Leipzig (Germany)	215	212	215	0.99987	1.0	65%	67%
	Northern Greece	103	98	102	0.999048	0.99981	66%	84%
	Hungary	103	100	103	0.999239	1.0	66%	83%

Group	Population	Number individuals	Number Yfiler haplotypes	Number RM Y-STR haplotypes	Yfiler haplotype diversity	RM Y-STR haplotype diversity	Yfiler Average % of differing loci	RM Y-STR Average % of differing loci
	Piemonte (Italy)	50	48	50	0.998367	1.0	64%	80%
	Marches (Italy)	99	99	98	1	0.999794	66%	81%
	Netherlands	112	112	112	1	1.0	61%	78%
	Warsaw (Poland)	364	333	363	0.998683	0.999985	58%	79%
	Wroclaw (Poland)	462	438	462	0.999634	1.0	58%	79%
	Sweden	292	279	289	0.999647	0.999929	65%	81%
	Switzerland	108	107	108	0.999827	1.0	63%	81%
Migrant Sub-Saharan Africa	British African	102	101	102	0.999806	1.0	60%	79%
	African American (Population 2)	249	246	248	0.999903	0.999968	65%	81%
	Danish Somali	91	64	89	0.983394	0.999512	44%	70%
Migrant Asian	British Asian	102	99	102	0.999418	1.0	69%	83%
	Hungarian Romani	103	77	101	0.991338	0.999619	62%	80%
Migrant European	US Hispanic	131	128	130	0.999648	0.999883	67%	82%
	US European	254	249	254	0.999844	1.0	61%	80%
	Australian European	167	166	167	0.999928	1.0	61%	81%
Biaka Pygmy		101	83	94	0.99505	0.998218	65%	83%

Supp. Tables S6, S7, and S8 are available as Excel files under the Supporting Information for this Article.

Supp. Table S6. RM Y-STR and Yfiler haplotypes obtained from the 12,272 individuals in 111 global populations

Supp. Table S7. Allele frequencies and ranges for individual populations, regional groups, and the complete dataset for each RM Y-STR marker

Supp. Table S8. RM Y-STR and Yfiler haplotypes for male relative pairs
